# Discrimination of indoor versus outdoor environmental state with machine learning algorithms in myopia observational studies

**DOI:** 10.1186/s12967-019-2057-2

**Published:** 2019-09-18

**Authors:** Bin Ye, Kangping Liu, Siting Cao, Padmaja Sankaridurg, Wayne Li, Mengli Luan, Bo Zhang, Jianfeng Zhu, Haidong Zou, Xun Xu, Xiangui He

**Affiliations:** 1 Department of Ophthalmology, Shanghai General Hospital, Shanghai Jiao Tong University, Shanghai Key Laboratory of Ocular Fundus Diseases, Shanghai Engineering Center for Visual Science and Photomedicine, Shanghai, China; 2 Department of Preventative Ophthalmology, Shanghai Eye Disease Prevention and Treatment Center, Shanghai Eye Hospital, Shanghai, China; 3 Peking-Tsinghua Center for Life Sciences, Academy for Advanced Interdisciplinary Studies, Center for Quantitative Biology (CQB), Peking University, Beijing, China; 4 Brien Holden Vision Institute, Sydney, NSW Australia; 5 School of Optometry and Vision Science, University of New South Wales, Sydney, NSW Australia; 6 Department of Maternal and Child Health, School of Public Health, Key Laboratory of Public Health Safety, Ministry of Education, Fudan University, Shanghai, China

**Keywords:** Machine learning algorithm, Smart watch, Outdoor time, Myopia intervention

## Abstract

**Background:**

Wearable smart watches provide large amount of real-time data on the environmental state of the users and are useful to determine risk factors for onset and progression of myopia. We aim to evaluate the efficacy of machine learning algorithm in differentiating indoor and outdoor locations as collected by use of smart watches.

**Methods:**

Real time data on luminance, ultraviolet light levels and number of steps obtained with smart watches from dataset A: 12 adults from 8 scenes and manually recorded true locations. 70% of data was considered training set and support vector machine (SVM) algorithm generated using the variables to create a classification system. Data collected manually by the adults was the reference. The algorithm was used for predicting the location of the remaining 30% of dataset A. Accuracy was defined as the number of correct predictions divided by all. Similarly, data was corrected from dataset B: 172 children from 3 schools and 12 supervisors recorded true locations. Data collected by the supervisors was the reference. SVM model trained from dataset A was used to predict the location of dataset B for validation. Finally, we predicted the location of dataset B using the SVM model self-trained from dataset B. We repeated these three predictions with traditional univariate threshold segmentation method.

**Results:**

In both datasets, SVM outperformed the univariate threshold segmentation method. In dataset A, the accuracy and AUC of SVM were 99.55% and 0.99 as compared to 95.11% and 0.95 with the univariate threshold segmentation (p < 0.01). In validation, the accuracy and AUC of SVM were 82.67% and 0.90 compared to 80.88% and 0.85 with the univariate threshold segmentation method (p < 0.01). In dataset B, the accuracy and AUC of SVM and AUC were 92.43% and 0.96 compared to 80.88% and 0.85 with the univariate threshold segmentation (p < 0.01).

**Conclusions:**

Machine learning algorithm allows for discrimination of outdoor versus indoor environments with high accuracy and provides an opportunity to study and determine the role of environmental risk factors in onset and progression of myopia. The accuracy of machine learning algorithm could be improved if the model is trained with the dataset itself.

## Background

Myopia is common all over the world, especially in East and South Asia. The prevalence of myopia in high school graduates may be as high as 80% to 90% with 10% to 20% of these individuals having high myopia (myopia worse than − 5.00 D) [1]. It is predicted that half of the population of the world will have myopia by 2050 [2], and one-tenth of the total population will have high myopia. Not only does myopia result in burden associated with the cost and management of the refractive error, the ocular complications resulting from high myopia are a significant cause of visual impairment and blindness [3, 4]. It has been suggested that the increasing prevalence of myopia can be largely explained by educational pressures resulting in long hours of near based activity and an associated reduction in outdoor time [5]. Evidence indicates that increased time outdoors has a positive effect on reducing the incidence of myopia as well as slowing the myopic shift in refractive errors [6–18].

To better understand the role of indoor and outdoor time on myopia incidence and prevalence, methods that can efficiently and objectively gather and accurately determine the indoor/outdoor location of the wearer as well as the time spent at these locations are needed. Presently, there are two methods that are actively used to gather such data. The first method utilizes subjective recall of time spent indoors versus outdoors with instruments such as telephone or face-to-face interviews, questionnaires, diaries and the like, and as such is subject to recall bias [3]. The second method relies on objective capture of data using for example, wearable devices or a biomarker. However, objective data gathering devices collect large amount of data and as such, are unwieldy to analyse using traditional techniques. Previously reported data with wearables calculated outdoor time using magnitude of sunlight exposure but the threshold used to discriminate between outdoor versus indoor environments varied between studies [4, 19–21]. In such studies, receiver operating characteristic (ROC) curves were drawn to obtain a cut-off point of sunlight exposure as the boundary to differentiate indoor versus outdoor environments. The area under the ROC curve (AUC) ranged from 0.82 to 0.96 but given they used a specific threshold suited for a particular environment, extrapolation of this threshold to other locations was not always possible. In addition, Guggenheim et al. [22] and Tideman et al. [23] attempted to apply biomarkers such as vitamin D and conjunctival ultraviolet autofluorescence (UVAF) levels [24, 25] to estimate sunlight exposure to obtain outdoor activity time. However, due to the invasiveness and complex nature of the procedure their use was limited, and therefore difficult to implement widely in the general public. More recently, other techniques were also used to collect information on time spent outdoors, such as the Global Positioning System (GPS) [26] and accelerometers [27–29].

To date, there have been no reports that have comprehensively considered multiple features to differentiate between indoor and outdoor environments. Methods used in artificial intelligence such as machine learning algorithms may be more effective in objectively determining the indoor/outdoor location of the users. We therefore applied machine learning algorithms to determine the accuracy of identifying and classifying outdoor and indoor environments for data collected with a smart watch (the wearable).

## Methods

### Smart watch

Our team designed and developed a smart watch named ‘Mumu’ equipped with a light sensor, accelerometer and GPS receiver. The light sensor samples luminance and ultraviolet intensity at 20-s intervals. Both the front and back of the smart watch have light sensors to detect whether it is being worn. The accelerometer consists of three axes that indicate the X, Y, and Z axes in space and through filtering, peak-valley detection, and removing interference, and finally converts these into counting steps. The built-in GPS receivers are used for receiving satellite signals and collecting data on the longitude and latitude of the location. Weather and temperature are synchronized in real time from the official website of the Shanghai Meteorological Bureau. The smart watch samples data once a minute. One piece of data consists of: time (year/month/day/00:00:00, 3 data points of luminance (lx), 3 data points on ultraviolet light intensity,count of steps, weather (sunny/cloudy) and wearing status. The above data were uploaded by the mobile terminal to a software platform, that was developed for collecting, analyzing, and counting the data.

### Data collection

Two datasets were collected and included: Dataset A (n = 76,284, 12 adults) and Dataset B (n = 23,539, 172 students from 3 schools). Each dataset consists of two parts. First, luminance, UV, number of steps and the weather were collected by the watch itself and transported to the computer terminal every minute. Second, the real positions were recorded by the volunteers or the supervisors every minute, and were uploaded to the computer terminal after summarizing and arranging. The research followed the tenets of the Declaration of Helsinki, the study was approved by the institutional review board of the Shanghai Jiao Tong University and informed consent obtained from all subjects after explanation of the nature and possible consequences of the study.

For Dataset A, we recruited 12 adults (23.8 ± 1.6 years, 21–28 years; 6 males and 6 females) with each adult wearing 2 smart watches (both the right and the left wrists) and sampling data from 3 scenes in a school (classroom, staircase, and playground) and 5 out-of-school scenes (park, house, square, road, and shopping mall) with data gathered on both sunny and cloudy days (all weather records were based on the real-time synchronization data from the official website of Shanghai Meteorological Administration). Additionally, time spent outdoors and indoors was recorded by the adult participants on a log sheet and taken to be the reference. A total of 76,284 pieces of data were uploaded to the software platform. A corresponding written log record of scene/location were considered for the analysis.

**Fig. 1:**
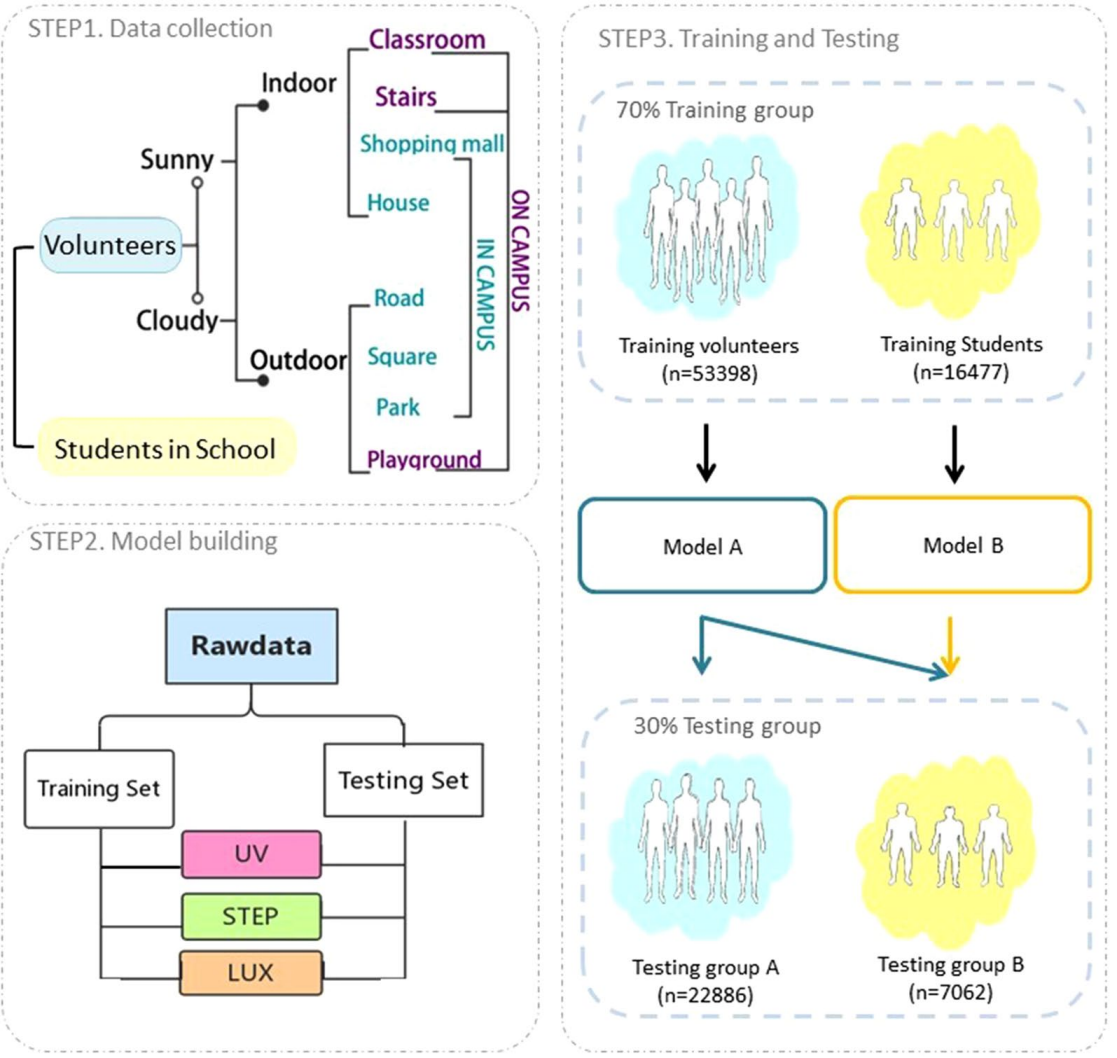
Flowchart of study design. In step 1, two datasets were collected. In step 2, each dataset was split into a training set to build a predicting model and a testing set to test the model. In step 3, two models were built and used to predict 2 testing groups

For Dataset B, we randomly chose 172 students (age 9–11 years) in 6 classes from three primary schools in Shanghai. Children wore the smart watches for one day at school, sampling data from 3 scenes in school (classroom, staircase and playground). The indoor or outdoor location of the students were recorded by twelve supervisors subjectively and recorded on a log sheet. The supervisors followed the students the entire day. A total of 23,539 data points were collected and uploaded to the software platform (Step 1 in Fig. 1).

### Machine learning algorithm

Discrimination of environment to either an indoor or an outdoor environment could be converted into a binary classification problem. In machine learning, the computer learns a decision boundary in the feature space that separates or classifies the data points into two classes. When the training is completed, the learning is transferred to classify new data points based on the learned decision boundary [30]. In binary classification, the most commonly used classification algorithms are neural network [31], support vector machine (SVM) [32], Gaussian process [33], random forest [34], naive Bayes [35], ensemble [36], and discriminant analysis [37]. Based on the comparison of seven kinds of algorithms, we chose support vector machine (SVM), as the tool to build the model due to its reported high accuracy. Table 1 showed seven common classification type deep learning algorithms to determine positional accuracy. Results reveal that all of the pairwise comparisons between these seven methods show significantly different (p < 0.001), except that between accuracy of neural network algorithm and average accuracy of these algorithms (p = 0.165).

**Table 1:** Common classification type deep learning algorithms to determine positional accuracy

Machine learning algorithms	Accuracy % (N)
Gaussian process	78.4% (17,949/22,886)
Ensemble	79.7% (18,242/22,886)
Neural network	80.2% (18,361/22,886)
Discriminant analysis	83.8% (19,183/22,886)
Naive Bayes	87.4% (20,006/22,886)
Random forest	90.9% (20,805/22,886)
SVM	97.1% (22,229/22,886)
Total	85.4% (136,775/160,202)

All of the pairwise comparisons between these seven methods show significantly different (p < 0.001), except that between accuracy of neural network algorithm and average accuracy of these algorithms (p = 0.165)

The core principle of the SVM algorithm is to establish a ‘hyperplane’ in the feature space that separates indoor and outdoor data by maximizing the distance between each of the data points from this hyperplane. In other words, firstly the algorithm involves finding the classification hyperplane. Thereafter, we adjusted the parameters which determined the hyperplane so that the distances from the data points to the separating hyperplane were maximized. Assuming we have ‘n’ points (x_i_, y_i_) in the training set, the parameters a_i_ and b can define the hyperplane. The hyperplane can be formulated as following.

$$f(x) = \sum\limits_{i = 1}^{n} {a_{i} y_{i} \left\langle {x_{i} ,x} \right\rangle + b}$$

where *x* indicates arbitrary vector sampling from the feature space. As the various data collected by smart watches are nonlinear, we added ‘kernel function’ to the model. That is, through the spatial transformation of φ (generally low-dimensional space is mapped to high-dimensional space x → φ (x)) to achieve nonlinear separation. Then the hyperplane defined in the transformed space (high-dimensional space) can be formulated as following.

$$f(x) = \sum\limits_{i = 1}^{n} {a_{i} y_{i} \left\langle {\phi (x_{i} ),\phi (x)} \right\rangle + b}$$

### Data processing

The data collected from the smart watches were integrated with the data as recorded by the participants and the supervisors. The valid data contained 11 features: time, luminance 1, luminance 2, luminance 3, ultraviolet intensity 1, ultraviolet intensity 2, ultraviolet intensity 3, counting steps, weather, wearing status and location but for the purpose of the analysis the following variables were used to build the SVM model: luminance 1, 2 and 3; ultraviolet intensity 1, 2 and 3 and counting steps.

### Model building

From each dataset, the processed data were separated into a training set (70% of the enrolled data) that was used to build the model, and a testing set (30% of the enrolled data) that was used to test the new model. For the procedure, we downloaded LIBSVM (A Library for Support Vector Machines), an SVM pattern recognition and regression package for windows [38], set up a Python environment on the computer and used ‘grid.py’ to optimize the parameters based on the processed data. ‘grid.py’ is a parameter selection program for C-SVM (Context-SVM) classification of RBF (Radial Basis Function) kernels. The user only needs to give a range of parameters, and ‘grid.py’ will use cross-validation to calculate the accuracy of each combination of parameters to find the best parameters. To optimize the model hyper-parameters, cross-validation was performed with different hyper-parameter settings in the training set. We used radial basis function (RBF) as the kernel function of our SVM model, which is expressed as

$$K(x,z) = e^{{ - \frac{{\left\| {x - z} \right\|^{2} }}{{2\gamma^{2} }}}}$$

in which γ is used to control the variance of RBF. The loss function we used to optimize the parameters was hinge loss with L2 regularization term, in which c controls the weights between hinge loss and L2 regularization as

$$L = \sum\limits_{i = 1}^{N} {[1 - y_{i} (wx_{i} + b)]_{ + } } + \frac{1}{2c}\left\| w \right\|^{2}$$

where w indicates the normal vector of the hyperplane of SVM algorithm which is also defined as

$${\text{w}} = \sum\limits_{i = 1}^{n} {a_{i} x_{i} y_{i} }$$

We tested 8000 paired of parameters γ and c to decide the best values for hyperparameters γand c. Finally, the SVM model was built using the generated parameters, and the training set data input into the program. Finally, we selected the luminance, ultraviolet, and count of steps as the characteristics based on the optimal feature combination given by the SVM model automatically. A further two SVM models were built: Model A from training group of Dataset A (n = 53,398) and Model B from training group of Dataset B (n = 16,477) (Step 2 in Fig. 1). Details of the python code can be found in Appendix.

### Location prediction

The SVM model predicted the indoor or outdoor location after inputting the testing group data.

We used both SVM Model A and traditional univariate threshold segmentation method to predict the indoor or outdoor location of testing group A (n = 22,886, 30% of Dataset A) and compared the accuracy, AUC, sensitivity, specificity and Youden Index of these two methods. Univariate threshold segmentation method drawn a receiver operator characteristics (ROC) curve to determine the best discriminating threshold for detection of indoor and outdoor activity and we chose luminance as a variable.

We then we applied Model A and univariate threshold segmentation method to predict the indoor or outdoor location of testing group B and compared the accuracy, AUC, sensitivity, specificity and Youden Index of the two methods in predicting the location of testing group B.

Finally, we applied SVM Model B and univariate threshold segmentation method to predict the indoor or outdoor location of testing group B (Step 3 in Fig. 1).

### Statistical analyses

Data were analyzed using SPSS version 22.0 (SPSS, Inc., Chicago, IL, USA). The luminance and UV values from different locations and weather conditions were tested using independent t-tests. The areas under the ROC curve with 95% confidence intervals were drawn to evaluate sensitivity, specificity and Youden Index of all data. The accuracy of the SVM machine learning algorithm compared with the real observation was assessed using Cohen’s kappa.

## Results

**Fig. 2:**
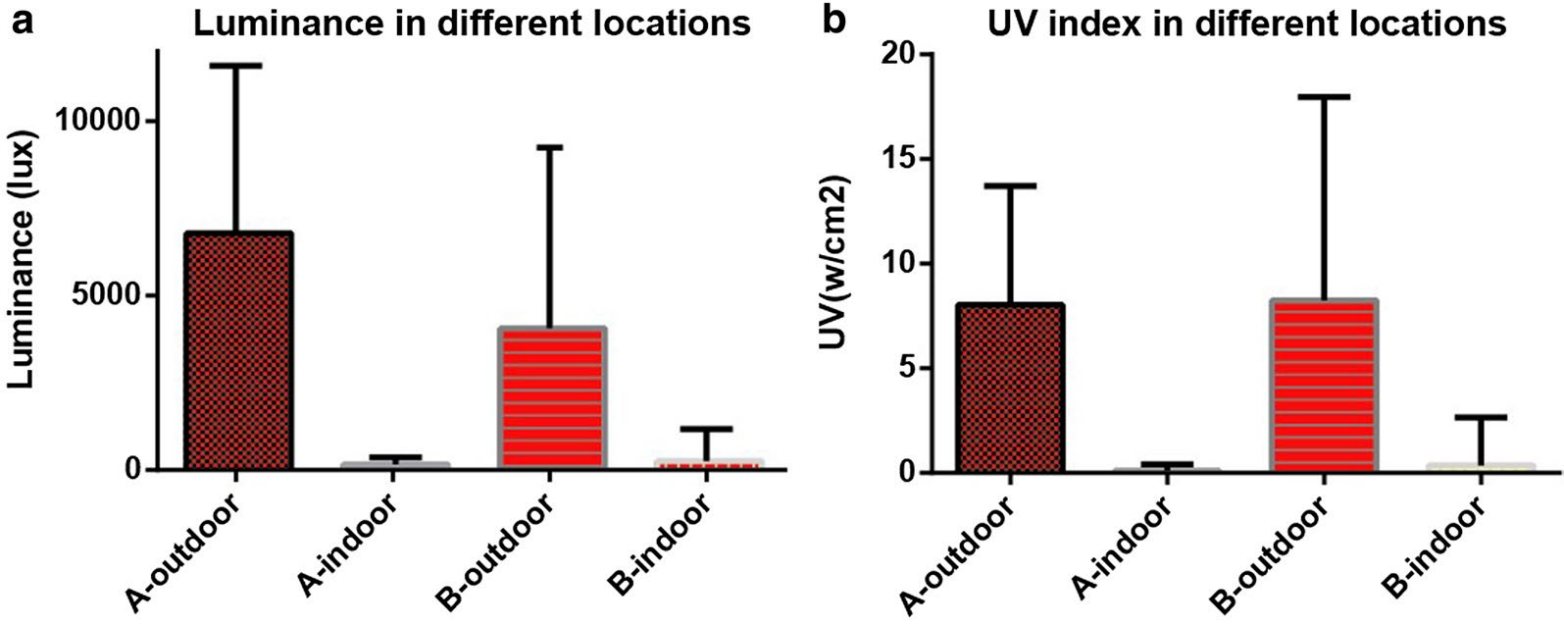
The luminance and UV of indoor and outdoor in dataset A and dataset B

Figure 2 presents the luminance and ultraviolet intensities as recorded using the smart watch from both datasets A and B. The total mean values of outdoor luminance and ultraviolet intensity was much higher than indoor luminance and ultraviolet intensity (p < 0.05). The absolute values of indoor luminance were relatively low (mean values lower than 400 lx), while those of outdoor illumination were relatively high (mean values higher than 1000 lx).

**Fig. 3:**
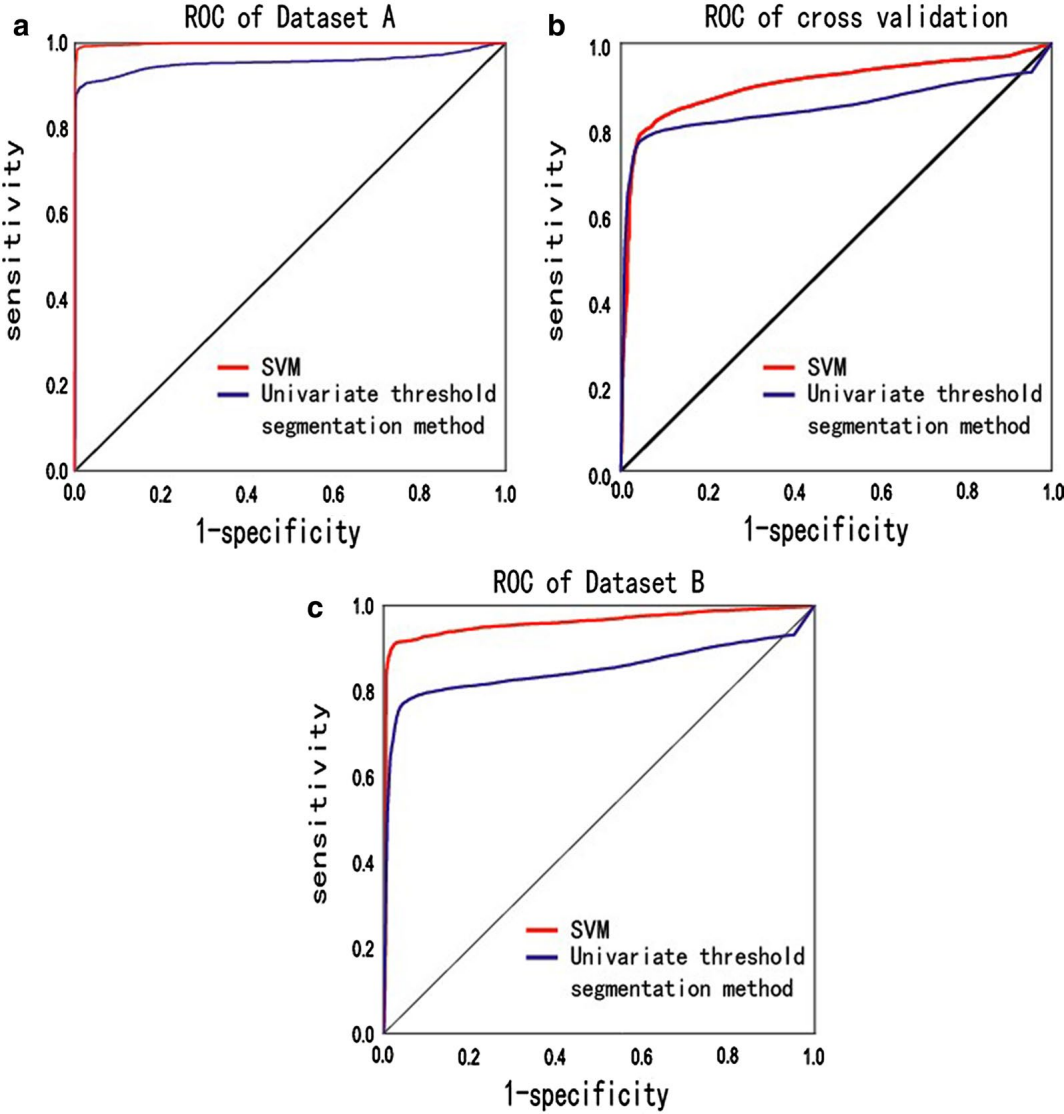
**a** The ROC curves of SVM (model A) and univariate threshold segmentation method for identifying indoor/outdoor locations of Testing group A. **b** The ROC curves of SVM (model A) and univariate threshold segmentation method for identifying indoor/outdoor locations of Testing group B. **c** The ROC curves of SVM (model B) and univariate threshold segmentation method for identifying indoor/outdoor locations of Testing group B

Based on the data collected, ROC curves for both the SVM and univariate threshold segmentation method were drawn for dataset A (Fig. 3a). The accuracy of SVM and univariate threshold segmentation were 99.55% and 95.11%. The AUCs of SVM and univariate threshold segmentation method were 0.99 and 0.95. The sensitivities of SVM and univariate threshold segmentation method were 0.99 and 0.89, respectively, and the specificities were 0.99 and 0.98 respectively.

In cross validation, ROC curves for SVM and univariate threshold segmentation method were drawn (Fig. 3b). The accuracy of SVM and univariate threshold segmentation method were 82.67% and 80.88%. The AUCs of SVM and univariate threshold segmentation method were 0.90 and 0.85. The sensitivities of SVM and univariate threshold segmentation method were 0.72 and 0.77, respectively, and the specificities were 0.97 and 0.95 respectively.

In dataset B, ROC curves for SVM and univariate threshold segmentation method were drawn (Fig. 3c). The accuracy of SVM and univariate threshold segmentation method were 92.44% and 80.88%. The AUCs of SVM and univariate threshold segmentation method were 0.96 and 0.85. The sensitivities of SVM and univariate threshold segmentation method were 0.89 and 0.77, respectively, and the specificities were 0.92 and 0.95 respectively.

Table 2 provides the results for the remainder 30% from set A as predicted by SVM Model A. Of the 22,886 data (7325 indoor, 15,561 outdoor), 102 (0.45%) were misclassified (59 outdoor locations were mistaken as indoors, and 43 indoor locations were mistaken as outdoors).

**Table 2:** Location of the testing group A predicted by Model A, the dataset B predicted by Model A and the testing group B predicted by Model B

Model	Data sets	Real location	Predicted		Total
			Outdoor	Indoor	
A	A	Outdoor	15,502	59^a^	15,561
		Indoor	43	7282	7325
		Total	15,545	7341	22,886
A	B	Outdoor	9799	3788^b^	13,587
		Indoor	291	9661	9952
		Total	10,090	13,449	23,539
B	B	Outdoor	4386	495^c^	4881
		Indoor	39	2142	2181
		Total	4425	2637	7062

^a^59 outdoor locations were mistaken as indoors, and 43 indoor locations were mistaken as outdoors Kappa = 0.990, p < 0.001

^b^3788 outdoor locations were mistaken as indoors, and 291 indoor locations were mistaken as outdoors. Kappa = 0.692, p < 0.001

^c^495 outdoor locations were mistaken as indoors, and 39 indoor locations were mistaken as outdoors. Kappa = 0.821, p < 0.001

Table 2 provides the results of locations of dataset B predicted by SVM Model A. Of the 23,539 data (9952 indoor, 13,587 outdoor), 4079 (17%) were misclassified (3788 outdoor locations were mistaken as indoors, and 291 indoor locations were mistaken as outdoors).

Table 2 provides the results of locations of dataset B predicted by SVM Model B. Of the 7062 data (2181 indoor, 4881 outdoor), 534 (7%) were misclassified (495 outdoor locations were mistaken as indoors, and 39 indoor locations were mistaken as outdoors).

## Discussion

With both datasets A and B, the SVM was more accurate than univariate method in predicting the outdoor location. However, when dataset A was used to predict dataset B, then the accuracy was lesser than when dataset B was used. Dataset A was collected by adult volunteers with good compliance. Therefore, the precision of data is high and the amount of data available is large. Dataset B was the real school data of primary school students. The wearers of the watches couldn’t record the true location by themselves, and therefore it was necessary for a supervisor to observe and record the real indoor and outdoor conditions one-to-one. In addition, students have normal curriculum arrangements, which is not convenient for intervention. So the amount of available data is small.

In previous studies, a single indicator (for example, luminance) was used to determine indoor and outdoor environments. Importantly, the luminance thresholds used to determine indoor versus outdoor environments varied across different studies, possibly due to the variations across the region, weather patterns, duration of data collection etc. This demonstrates that the method of using a single indictor with a cut-off threshold as basis for determination may not apply well in a real-life, long term monitoring situation. For example, our study found that the luminance outdoors on cloudy days was lower than that on sunny days. A predictive model output using data gathered from sunny days alone would likely have a higher cut-off threshold for classification of outdoor versus indoor locations. GPS was another method used to detect location through comparing the signal-to-noise ratio characteristics of indoor and outdoor environments. Tandon [20] found that a threshold of an SNR > 250 can distinguish indoor and outdoor environments (sensitivity = 82%, specificity = 88%, Youden Index = 0.70 and AUC = 0.890), which was lower than the light sensor method reported by Jennifer et al. [21]. In the current study, we applied a machine learning algorithm, to differentiate between indoor and outdoor environments for data on multiple environmental features collected from a smart watch. The predictive performance of the machine learning algorithm was satisfactory and provides an alternative opportunity to objectively detect and record time spent outdoors by children and adolescents. Application of machine learning algorithms has greatly contributed to medical data classification.

In our study, machine learning was used to convert the indoor and outdoor discrimination problem into a data classification problem. Multiple factors were taken into consideration, including time, illumination, ultraviolet intensity and counted steps. Overall considerations and weigh comprehensively of our methods design is more suitable for the actual situation. The SVM algorithm showed the best performance among seven candidate machine learning algorithms in our study. We compared the SVM algorithm with other published methods, including light sensors and GPS (Table 3) and it is observed that the SVM algorithm has higher sensitivity (99%), specificity (99%) and Youden Index (0.99) compared to other methods. Thus, the SVM algorithm has the potential to be a more reliable and feasible tool for separating indoor and outdoor environments using multiple dimensions instead of one dimension. Moreover, in order to more accurately predict location by taking advantage of multiple variable analysis, our approach can use not only numerical variables but also categorical variables by converting the categorical input to numerical input. With an appropriate kernel, our algorithm works well even if the data were not linearly separable in the base feature space, making the model match the actual circumstances better and being more accurate than previous studies.

**Table 3:** Machine learning algorithm compared with other published methods

Author	Method	Cut point	AUC	Sensitivity	Specificity	Youden Index
Tandon [20]	Luminance segmentation method	110 lx	0.82	74%	86%	0.60
Tandon [20]	GPS segmentation method	250 SNR^a^	0.89	82%	88%	0.70
Flynn [21]	Luminance segmentation method	240 lx	0.96	92%	90%	0.82
Dharani [19]	Luminance segmentation method	1000 lx	–	–	–	–
In our study	SVM Machine learning algorithm	–	0.99	99%	99%	0.99

^a^Signal-to-noise ratio (SNR); Area under the ROC curve (AUC)

However, our study had some limitations. Firstly, the amount of data collected in Dataset B is small, because the collection requirements were difficult and the number of supervisors were insufficient. Secondly, the data were collected only on sunny and cloudy days. Other weather conditions, such as rainy, snowy and foggy, should be added to the learning pool of the SVM model. Finally, the scenes selected were limited to 3 scenes (classroom, playground, and stairs) in a primary school and 5 out-of-school scenes (park, road, square, house, and shopping mall). Although they reflected the most frequent scenes in a school-age child’s daily life, more scenes are needed to improve the applicability of this method.

The collection of big data from an individual’s daily life provides a good platform for the application and development of artificial intelligence for the benefits of public health. Importantly, such data are more valid as they are not limited to hospital diagnostic information or radiologic history but are generated though the course of daily life and therefore are more representative of the individual’s state. With such data, an individual can make a more valid and accurate assessment of their personal health status and the data will provide insights to disease development and therefore prevention patterns. Clearly, the use of appropriate algorithms to harness the data to meaningful conclusions is critical. Having considered the above, we believe that the machine learning algorithm we applied could make smart watch more intelligent to distinguish indoor between outdoor and record outdoor time precisely and is useful as an objective and feasible device for exploring specific relations between myopia and outdoor time. Now we have applied this method in our outdoor intervention clinical trail from 2017 [39].

## Conclusion

Machine learning algorithm allows for discrimination of outdoor versus indoor environments with high accuracy and provides an opportunity to study and determine the role of environmental risk factors in onset and progression of myopia. Furthermore, the smart watch in combination with the machine learning algorithm could provide a useful monitoring tool for community- or school-based public health interventions or individual health management.

## Data Availability

All data generated or analyzed during this study are included in this published article and its additional information files.

## Abbreviations

SVM: support vector machine
ROC: receiver operating characteristic
AUC: area under the curve
UVAF: ultraviolet autofluorescence
GPS: Global Positioning System
LIBSVM: a library for support vector machines
C-SVM: context-SVM
RBF: radial basis function

## References

[1] Morgan IG, Ohno-Matsui K, Saw SM (2012). Myopia. Lancet.

[2] Holden BA, Fricke TR, Wilson DA, Jong M, Naidoo KS, Sankaridurg P (2016). Global prevalence of myopia and high myopia and temporal trends from 2000 through 2050. Ophthalmology.

[3] Wu L, Sun X, Zhou X, Weng C (2011). Causes and 3-year-incidence of blindness in Jing-An District, Shanghai, China 2001–2009. BMC Ophthalmol.

[4] Xu L, Wang Y, Li Y (2006). Causes of blindness and visual impairment in urban and rural areas in Beijing: the Beijing Eye Study. Ophthalmology.

[5] Morgan IG, French AN, Ashby RS (2018). The epidemics of myopia: aetiology and prevention. Prog Retin Eye Res.

[6] Xiong S, Sankaridurg P, Naduvilath T (2017). Time spent in outdoor activities in relation to myopia prevention and control: a meta-analysis and systematic review. Acta Ophthalmol.

[7] Wu PC, Chen CT, Lin KK (2018). Myopia prevention and outdoor light intensity in a school-based cluster randomized trial. Ophthalmology.

[8] He M, Xiang F, Zeng Y, Mai J, Chen Q, Zhang J, Smith W, Rose K, Morgan IG (2015). Effect of time spent outdoors at school on the development of myopia among children in China: a randomized clinical trial. JAMA.

[9] Sherwin JC, Reacher MH, Keogh RH, Khawaja AP, Mackey DA, Foster PJ (2012). The association between time spent outdoors and myopia in children and adolescents: a systematic review and meta-analysis. Ophthalmology.

[10] Dirani M, Tong L, Gazzard G, Zhang X, Chia A, Young TL, Rose KA, Mitchell P, Saw SM (2009). Outdoor activity and myopia in Singapore teenage children. Br J Ophthalmol.

[11] French AN, Ashby RS, Morgan IG, Rose KA (2013). Time outdoors and the prevention of myopia. Exp Eye Res.

[12] Wu PC, Tsai CL, Wu HL, Yang YH, Kuo HK (2013). Outdoor activity during class recess reduces myopia onset and progression in school children. Ophthalmology.

[13] Wu PC, Tsai CL, Hu CH, Yang YH (2010). Effects of outdoor activities on myopia among rural school children in Taiwan. Ophthalmic Epidemiol.

[14] Guo Y, Liu LJ, Xu L, Lv YY, Tang P, Feng Y, Meng M, Jonas JB (2013). Outdoor activity and myopia among primary students in rural and urban regions of Beijing. Ophthalmology.

[15] Lin Z, Vasudevan B, Jhanji V, Mao GY, Gao TY, Wang FH, Rong SS, Ciuffreda KJ, Liang YB (2014). Near work, outdoor activity, and myopia in children in rural China: the Handan offspring myopia study. BMC Ophthalmol.

[16] Guggenheim JA, Northstone K, McMahon G, Ness AR, Deere K, Mattocks C, St Pourcain B, Williams C (2012). Time outdoors and physical activity as predictors of incident myopia in childhood: a prospective cohort study. Investig Ophthalmol Vis Sci.

[17] Jin JX, Hua WJ, Jiang X, Wu XY, Yang JW, Gao GP, Fang Y, Pei CL, Wang S, Zhang JZ, Tao LM (2015). Effect of outdoor activity on myopia onset and progression in school-aged children in northeast China: the Sujiatun Eye Care Study. BMC Ophthalmol.

[18] Guo Y, Liu LJ, Xu L, Tang P, Lv YY, Feng Y, Meng M, Jonas JB (2013). Myopic shift and outdoor activity among primary school children: one-year follow-up study in Beijing. PLoS ONE.

[19] Dharani R, Lee C-F, Theng ZX, Drury VB, Ngo C, Sandar M, Wong T-Y, Finkelstein EA, Saw S-M (2012). Comparison of measurements of time outdoors and light levels as risk factors for myopia in young Singapore children. Eye.

[20] Tandon PS, Saelens BE, Zhou C, Kerr J, Christakis DA (2013). Indoor versus outdoor time in preschoolers at child care. Am J Prev Med.

[21] Flynn JI, Coe DP, Larsen CA, Rider BC, Conger SA, Bassett DR (2014). Detecting indoor and outdoor environments using the ActiGraph GT3X + light sensor in children. Med Sci Sports Exerc.

[22] Guggenheim JA, Williams C, Northstone K, Howe LD, Tilling K, St PB (2014). Does vitamin D mediate the protective effects of time outdoors on myopia? Findings from a prospective birth cohort. Investig Ophthalmol Vis Sci.

[23] Tideman JW, Polling JR, Voortman T, Jaddoe VW, Uitterlinden AG, Hofman A (2016). Low serum vitamin D is associated with axial length and risk of myopia in young children. Eur J Epidemiol.

[24] Sherwin JC, Hewitt AW, Coroneo MT, Kearns LS, Griffiths LR, Mackey DA (2012). The association between time spent outdoors and myopia using a novel biomarker of outdoor light exposure. Investig Ophthalmol Vis Sci.

[25] Sherwin JC, McKnight CM, Hewitt AW, Griffiths LR, Coroneo MT, Mackey DA (2012). Reliability and validity of conjunctival ultraviolet autofluorescence measurement. Br J Ophthalmol.

[26] Wu J, Jiang C, Jaimes G, Bartell S, Dang A, Baker D (2013). Travel patterns during pregnancy: comparison between Global Positioning System (GPS) tracking and questionnaire data. Environ Health.

[27] Pearce M, Page AS, Griffin TP, Cooper AR (2014). Who children spend time with after school: associations with objectively recorded indoor and outdoor physical activity. Int J Behav Nutr Phys Act.

[28] Cooper AR, Page AS, Wheeler BW, Hillsdon M, Griew P, Jago R (2010). Patterns of GPS measured time outdoors after school and objective physical activity in English children: the PEACH project. Int J Behav Nutr Phys Act.

[29] Webber SC, Porter MM (2009). Monitoring mobility in older adults using global positioning system (GPS) watches and accelerometers: a feasibility study. J Aging Phys Act.

[30] Baştanlar Y, Özuysal M (2014). Introduction to machine learning. miRNomics: microRNA biology and computational analysis.

[31] Hagan MT, Demuth HB, Beale MH (1996). Neural network design.

[32] Joachims Thorsten (1998). Text categorization with Support Vector Machines: Learning with many relevant features. Machine Learning: ECML-98.

[33] Rasmussen CE (2004). Gaussian processes in machine learning. Advanced lectures on machine learning.

[34] Liaw A, Wiener M (2002). Classification and regression by randomForest. R News.

[35] McCallum A, Nigam K. A comparison of event models for naive bayes text classification. In: AAAI-98 workshop on learning for text categorization, vol. 752, no. 1. 1998. p. 41–8.

[36] Dietterich Thomas G. (2000). Ensemble Methods in Machine Learning. Multiple Classifier Systems.

[37] Fraley C, Raftery AE (2002). Model-based clustering, discriminant analysis, and density estimation. J Am Stat Assoc.

[38] Hsu CW, Chang CC, Lin CJ. A practical guide to support vector classification. 2003. p. 1–16.

[39] He X, Sankaridurg P, Xiong S (2019). Shanghai time outside to reduce myopia trial: and baseline data. Clin Exp Ophthalmol..

